# Cross-drug resistance to sunitinib induced by doxorubicin in endothelial cells

**DOI:** 10.3892/ol.2014.2819

**Published:** 2014-12-22

**Authors:** LIMIN HUANG, CHAOQUAN HU, MÉLANIE DI BENEDETTO, RÉMI VARIN, JIELIN LIU, JIAN JIN, LI WANG, JEAN-PIERRE VANNIER, ANNE JANIN, HE LU, HONG LI

**Affiliations:** 1 Department of Oncology, People’s Hospital of Guizhou Province, Guiyang, Guizhou 550000, P.R. China; 2 Department of Surgery, Affiliated Hospital of Guiyang Medical University, Guiyang, Guizhou 550004, P.R. China; 3 French Institute of Health and Medical Research, UMR-S 1165, University Institute of Hematology, Saint Louis Hospital, Paris 75010, France; 4 Laboratory of MERCI (EA 3829), Faculty of Medicine and Pharmacy, University of Rouen, Rouen 76183, France; 5 School of Medicine and Pharmaceutics, Jiangnan University, Wuxi, Jiangsu 214122, P.R. China; 6 Laboratory of Pathology, Paris Diderot University, Sorbonne Paris Cité, UMR-S 1165, France; 7 Saint-Louis Hospital, Laboratory of Pathology, Paris 75010, France

**Keywords:** drug resistance, endothelial cells, cancer therapy, ABC family, sunitinib

## Abstract

Multiple drug resistance remains an unsolved problem in cancer therapy. A previous study has demonstrated that the chemotherapeutic drug doxorubicin (Dox) induced upregulation of P-glycoprotein in endothelial cells, resulting in a 20-fold increase in drug resistance and reduced efficiency of doxorubicin treatment in a mouse tumor model. In the present study, the cross-resistance and sensitivity of HMECd1 and HMECd2 established cell lines to anti-angiogenic drugs, particularly sunitinib, was explored. The results revealed that Dox treatment induced a significant increase in the breast cancer resistance protein (ABCG2) gene transcription and protein expression. This increase gave rise to a 4- to 5-fold increase in the half maximal inhibitory concentration of the HMECd1 and HMECd2 cells in response to sunitinib treatment *in vitro*. Functionally, the role of ABCG2 in the resistance to sunitinib was confirmed by the use of the ABCG2 inhibitors fumitremorgin C and diethylstilbestrol, which blocked cell resistance. The present study indicates that endothelial cells exhibit cross-resistance between cytotoxic drugs and anti-angiogenic drugs. This suggests that multiple drug resistance induced by chemotherapy in endothelial cells may affect the efficiency of anti-angiogenic drugs.

## Introduction

Cancer development depends on a complex tissue environment for growth and metastasis (1). It is now widely recognized that immune reaction and vascular growth support tumor growth (2). Since that breakthrough, anti-angiogenic therapy has been successfully introduced in clinical cancer therapy to starve tumors (3–6).

Theoretically, using anti-angiogenic agents to block factors crucial to tumor angiogenesis should offer several advantages over conventional chemotherapy agents. First, anti-angiogenic therapy can treat all solid tumors without being restricted to a specific tumor type. Second, as anti-angiogenic therapy targets the endothelial cells within the tumor vasculature, the agents within the blood stream directly affect the targeted cells, without penetration of the tumor being necessary. Furthermore, anti-angiogenic drugs are not expected to give rise to drug resistance as they do not target the highly mutable cancer cell population, but rather the more genetically stable endothelial cells (7,8). Anti-angiogenic therapy should thus allow for prolonged treatment with anti-angiogenic drugs, without giving rise to resistance.

Currently, hundreds of clinical trials involving anti-angiogenic agents are underway. Despite the initial promising performance of anti-angiogenic drugs in clinical trials, anti-angiogenesis therapy faces numerous challenges, including inherent and acquired resistance. The majority of cancer patients eventually demonstrate a lack of response to anti-angiogenic therapy while on the treatment regimen (4–6). Studies using clinical and preclinical models have documented the involvement of certain molecular and cellular mechanisms (9–11). At present, the proposed mechanisms include alternative angiogenic pathways, such as selective pressure of hypoxia, cancer stem cells, autophagy, recruitment of vascular progenitors and modulators, and tumor dormancy (11). In particular, previous studies indicate that acquired drug resistance in tumor endothelial cells is involved in drug resistance in cancer patients (12,13).

Multidrug resistance is considered a major obstacle for successful chemotherapy in the treatment of cancer. Chemotherapy loses effectiveness over time. One reason is the drug resistance caused by the compensatory response of tumor cells (10,14). One of the main underlying mechanisms for multidrug resistance in cancer chemotherapy is the overproduction of ATP-binding cassette (ABC) transporter, which serves as a pump to remove toxic drugs from tumor cells, thus rendering the tumor cells resistant to multiple chemotherapeutic drugs.

Clinical studies of human cancer have found a correlation between P-glycoprotein (P-gp) overexpression in tumor tissues with decreased survival and poor prognosis (14). High P-gp expression has been found in tumor endothelial cells, likely in response to vascular endothelial growth factor stimulation (15). We have also shown that the chemotherapeutic agent doxorubicin (Dox) induces high levels of P-gp in endothelial cells (12). Our previous study established two endothelial cell lines, HMECd1 and HMECd2, that exhibited high drug resistance to doxorubicin (Dox) induction *in vitro*. These two stabilized sub cell lines demonstrated 15- and 24-fold increases in resistance to Dox. Acquired drug resistance in endothelial cells was also revealed to attenuate the efficacy of doxorubicin treatment in a mouse tumor model. Dox-induced drug resistance in these endothelial cells was predominantly due to MDR1/P-gp upregulation. Inhibiting the activity of P-gp could reverse the resistance of endothelial cells. Furthermore, the drug resistance of endothelial cells attenuated the efficacy of doxorubicin treatment *in vivo* (12). This previous study indicated that the acquired drug resistance of tumor vessels plays a critical role in cancer therapy.

The breast cancer resistance protein (ABCG2) is another ABC transporter that has been identified as a molecular cause of multidrug resistance in diverse cancer cells (16,17). As an efflux transporter for xenobiotics and unwanted toxic compounds, ABCG2 has been characterized as an important component of self-defense systems in organisms (18). In the brain microvasculature, ABCG2 is located on the luminal surface of microvessel endothelium and hence may constitute an important component of the blood-brain barrier (19).

Sunitinib is an oral multi-targeted receptor tyrosine kinase inhibitor of vascular endothelial growth-factor receptors (20,21). Currently, sunitinib is used to treat advanced or metastatic renal cell carcinoma, gastrointestinal stromal tumors, meningioma and pancreatic neuroendocrine tumors. Clinical trials of combined sunitinib therapy with chemotherapy are ongoing (22–24). Patient resistance to sunitinib treatment has been reported (11,25,26). The aim of the present study was to investigate the risk of acquired and cross-resistance to anti-angiogenic drugs in endothelial cells during chemotherapy.

## Materials and methods

### Materials

Mouse monoclonal anti-P-gp, anti-ABCG2 and anti-MRP1 antibodies were purchased from Abcam (Cambridge, UK). Sunitinib was obtained from Pfizer, Inc. (New York, NY, USA). Doxorubicin chlorhydrate was purchased from Amersham Pharmacia Biotech, Inc. (Uppsala, Sweden). Verapamil was obtained from Calbiochem (Billerica, MA, USA). Paclitaxel, vinblastine, cyclosporine A, fumitremorgin C, diethylstilbestrol and MK571 were purchased from Sigma-Aldrich (Saint Louis, MO, USA).

### Cell culture

Parental and resistant HMEC-1 cell lines, obtained from Dr TL Lawley (Department of Dermatology, Atlanta, GA, USA), were cultured in MCDB-131 medium supplemented with 10% fetal calf serum (FCS), 2 mM L-glutamine, 10 ng/ml epidermal growth factor, 1 μg/ml hydrocortisone, 100 units/ml penicillin, and 100 μg/ml streptomycin, as described elsewhere (12,27). Dox-resistant HMEC cells were obtained by continuously exposing cells to increasing concentrations of Dox, between 0.001 and 0.24 μg/ml, over a 12-week period, as previously described (12). Two sub cell lines of HMEC-1 cells were collected, HMECd1 cells were maintained in a culture with 0.08 μg/ml Dox and HMECd2 cells were maintained in 0.24 μg/ml Dox. No mutagenic agents were used to establish these Dox-resistant HMEC cells. To observe the reversibility of the drug resistance of the cells, Dox was withdrawn from the culture medium of HMECd1 and HMECd2 cells. All cell types were digested with trypsin-EDTA once or twice a week and cultured in a 37°C incubator with a 100% humidified atmosphere of 5% CO_2_.

### MTS cell proliferation assay

Cell viability was determined using MTS cell proliferation assay (Promega, Madison, WI, USA). Cells grew to a confluence of 90% in 75 cm^2^ cell culture flasks and were passed into 96-well plates (7,500 cells/well). Each well contained 100 μl of culture medium, which was supplemented with various concentrations of drugs or with a concentration of dimethyl sulfoxide as a control. Following incubation for either 24, 48 or 72 h, 20 μl of the MTS reagent was added to each well and the plate was placed in the 5% CO_2_ incubator at 37°C for an additional 2 h. The optical density (OD) was then read at 492 nm using a microplate reader (Labsystems Multiskan MS; MTX Lab Systems Inc., Vienna, VA, USA). The half maximal inhibitory concentration (IC_50_) values were defined as the concentration of drug producing 50% inhibition of cell growth and the resistance index corresponding to the ratio of IC_50_ values between the resistant and parental cell lines. Experiments were performed in triplicate and repeated at least three times.

### Blocking effect assay

The experiments used ABCG2 inhibitors, 5 μM fumitremorgin C and 0.5 μM diethylstilbestrol, and P-gp inhibitors, 2.5 μM cyclosporine A, 1 μM verapamil and 5 μM MK571. Following incubation for 48 or 72 h, the cell viability was assessed using an MTS assay. The reversal fold (RF) values, a measure of the potency of reversal, were obtained by fitting the data to RF = IC_50_ of cytotoxic drug alone/IC_50_ of cytotoxic drug in the presence of a modulator (28).

### Evaluation of mRNA expression via quantitative polymerase chain reaction (qPCR)

The HMEC-1, HMECd1 and HMECd2 cells were treated with 2.5 μM cyclosporine A, 1 μM verapamil, 5 μM fumitremorgin C, 0.5 μM diethylstilbestrol or 5 μM MK571 for 24 h. Subsequent to incubation, the treated and non-treated cells were harvested, and total RNA was prepared using the SV total RNA isolation system kit (Promega). The purity of total RNA was checked by a ratio of A_260_/A_280_ (>1.9). Total RNA (50 ng) was used to synthesize the first-strand cDNA in a 20-μl reaction solution using the GoScript Reverse Transcription System kit (Promega). Then, 2 μl of cDNA was used for qPCR in triplicates using a TaqMan^®^ gene expression assay (Applied Biosystems, Foster City, CA, USA) and the primers for P-gp (Hs01067802_m1), ABCG2 (Hs01053790_m1), multidrug resistance protein (MRP) 1 (Hs00219905_m1), as well as the primers for TATA box binding protein (TBP) as controls (Hs99999910_m1; Applied Biosystems). qPCR was performed by 10 min of initial denaturation followed by 44 cycles of 15 sec at 95°C and 60 sec at 60°C in a BioRad CFX96® Real-time system (Bio-Rad Laboratories, Hercules, CA, USA). The ΔCt method was used to analyze the qPCR results, and TBP was used as an internal control for mRNA-level normalization.

### Evaluation of protein expression using western blot analysis

Western blot analysis was performed on whole-cell lysates by incubating the cells in the lysis buffer (10 mM Tris pH 6.8, 1 mM EDTA, 10% Nonidet P-40, 1 mM phenylmethanesulfonyl fluoride, 0.1% SDS) on ice for 30 min. Cell debris was removed by centrifugation at 16,000 × g for 10 min. Protein concentration was determined by bicinchoninic acid protein assay (Thermo Fisher Scientific, Waltham, MA, USA). A 50 μg protein sample from each sample was loaded on an 8% SDS-PAGE gel, and the protein was transferred to a polyvinylidene fluoride membrane using the iBlot dry blotting system (Invitrogen, Carlsbad, CA, USA). The membranes were blocked with 5% non-fat dry milk for 1 h and incubated with either anti-P-gp (ab-3364; 1:20; Abcam), anti-MRP1 (ab-32574; 1:250; Abcam) or anti-ABCG2 antibodies (ab-3380; 1:100; Abcam) at 4°C overnight. The membranes were then washed with a Tris buffered saline with Tween 20 buffer for 1 h and incubated with the appropriate horseradish peroxidase-conjugated secondary antibodies (Invitrogen), diluted in blocking buffer, for 1 h at room temperature. Subsequent to washing, western blotting luminol reagent (Santa Cruz Biotechnology, Inc., Dallas, TX, USA) was added to the membranes and the chemiluminescence was recorded using a Fuji LAS-3000 system (Fujifilm, Tokyo, Japan). The membranes were then treated with an antibody-stripping buffer (Gene Bio-Application Ltd., Kfar Hanagid, Israel) and incubated with anti-actin antibody (1:4,000 dilution; Sigma-Aldrich) as a control.

### Statistical analyses

The data were analyzed using one-way analysis of variance and Mann-Whitney U tests, as appropriate. The qPCR data is presented as the mean ± standard error of the mean. The remaining data is presented as the mean ± standard deviation. P≤0.05 was considered to indicate a statistically significant difference.

## Results

### Endothelial cells resistant to anti-angiogenesis drugs

To address the question of the response of HMECd1 and HMECd2 to the anti-angiogenic drugs, the efficacy of sunitinib was tested *in vitro.* The first experiment with MTS assay revealed that HMEC-1 cells are initially sensitive to sunitinib treatment. However, compared with their parental cells, HMECd1 and HMECd2 cells revealed 2.1- and 4.51-fold increases in drug-resistance to sunitinib (Table I). The increase in sunitinib resistance accompanied the increase in Dox resistance (Table I). This observation corresponded with the typical multi-drug resistance of endothelial cells in response to Dox induction.

### ABCG2 and P-gp are predominantly expressed in the resistant endothelial cells

**Figure 1:**
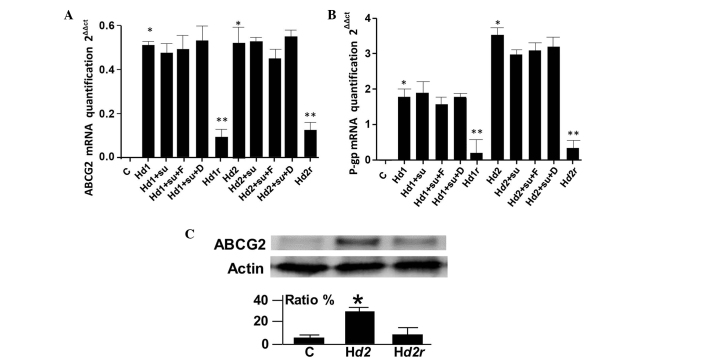
Induced ABCG2 expression in HMEC-1 endothelial cells. (A) qPCR (primer, Hs01053790_m1) of ABCG2 mRNA levels in treated or non-treated HMEC-1, HMECd1, HMECd2, HMECd1r and HMECd2r cells cultured without Dox for three weeks. Sunitinib, fumitremorgin C and diethylstilbestrol were used to treat the cells. The results were obtained from three independent experiments. ^*^P<0.05, vs. non-treated cells. (B) qPCR (primer, Hs01067802_m1) of P-gp mRNA levels in treated or non-treated HMEC-1, HMECd1, HMECd2 and HMECd2r cells. Sunitinib, fumitremorgin C, and diethylstilbestrol were used to treat the cells. The results were obtained from three independent experiments. ^*^P<0.05 and ^**^P<0.01, vs. non-treated cells. (C) Western blot analysis of ABCG2 levels in HMEC-1, HMECd2 and HMECd2r cells. The data for the ratio were obtained from three repeated blots. ^*^P<0.05, vs. the control and Hd2r cells. C, HMEC-1; Hd1, HMECd1; Hd2, HMECd2; Hd1r, HMECd1r; Hd2r, HMECd2r; Su, sunitinib; F, fumitremorgin C; D, diethylstilbestrol; ABCG2, breast cancer resistance protein.

qPCR was used to measure changes in drug efflux transporter gene expression in the Dox-induced resistant endothelial cells. The P-gp and ABCG2 expression in HMECd1 and HMECd2 cells increased significantly compared to parental cells (1.41- and 1.68-fold for ABCG2; 3.4- and 7.2-fold for P-gp; Fig. 1). To evaluate the influence of the ABC transporter blockers used in the study of gene expression, the changes in P-gp, ABCG2 and MRP1 mRNA levels in the presence of the inhibitors of the three transporters were quantified, respectively.

There was no significant change in the gene expression of P-gp and ABCG2 in HMECd1 and HMECd2 cells when the function of P-gp or ABCG2 was blocked (Fig. 1A and B). The qPCR results also indicated that MRP1 was not induced in HMECd1 and HMECd2 cells (data not shown). Western blotting revealed approximately two- to four-fold increases in the ABCG2 protein expression of the cells. Withdrawal of Dox in the culture media for more than three weeks resulted in a decrease in P-gp and ABCG2 expression in HMECd1 (Hd1r)and HMECd2 (Hd2r) cells (Fig. 1). This indicated that the P-gp and ABCG2 expression was reversible.

### Blocking ABCG2 attenuates the resistance of Dox-induced endothelial cells to sunitinib

The survival of HMECd1 and HMECd2 cells was examined subsequent to sunitinib treatment in the presence of the P-gp blockers cyclosporine A and verapamil, or in the presence of the ABCG2 blockers fumitremorgin C and diethylstilbestrol. The results revealed that only the blockade of ABCG2 function significantly restored the sensitivity of Dox-induced of endothelial cells to sunitinib (Table II). By contrast, the P-gp inhibitors demonstrated no such effect.

## Discussion

Drug resistance remains a difficult, unsolved issue in cancer therapy. Since the start of the use of anti-angiogenic therapy, it was expected to be an exception to drug resistance (7,8). However, primary or acquired drug resistance was soon reported in anti-angiogenic therapy and the mechanisms of that resistance have been periodically reviewed (9–11). In a previous study, it was demonstrated that doxorubicin successfully induced multiple resistance in endothelial cells (12). In the present study, the response of two stabilized endothelial cell lines, HMECd1 and HMECd2, to the anti-angiogenesis drug sunitinib was evaluated.

The present study provides evidence that ABCG2 mediates the substrate efflux of sunitinib in endothelial cells. The present study demonstrated that, in addition to P-gp upregulation, ABCG2 expression was upregulated in HMECd1 and HMEd2 cells. The ABCG2 protein levels, revealed by western blot analysis, were found to possess a two- to four-fold increase in HMECd1 and HMECd2 cells compared with their parental cells. Similarly, qPCR revealed 1.41- and 1.68-fold increases in ABCG2 gene expression in the HMECd1 and HMECd2 cells. It was revealed that P-gp was not involved in sunitinib resistance as two inhibitors of P-gp, cyclosporine A and verapamil, failed to reverse sunitinib resistance in HMECd1 and HMEC2. By contrast, the blockade of ABCG2 activity by fumitremorgin C and diethylstilbestrol greatly inhibited the capacity of the cells for sunitinib resistance. These results indicate that ABCG2 plays a major functional role in the resistance of HMECd1 and HMEC2 endothelial cells to sunitinib *in vitro*. The present results were in agreement with previous reports that revealed the involvement of ABCG2 in the resistance of cells to sunitinib treatment, although the involvement of P-gp has not been excluded in *in vivo* studies (29–31). As resistance to sunitinib in endothelial cells can be induced by Dox, potential cross-resistance in combined therapy that uses chemotherapy and targeted therapy may occur in clinical trials.

Cross-resistance in cancer therapy was observed in clinical settings >50 years ago (32,33). Currently, as the combined use of chemotherapy and anti-angiogenic drugs develops rapidly, it is particularly important to explore cross-resistance. Indeed, clinical trials evaluating the combined use of targeted therapy and chemotherapy are extremely dynamic. For example, >10 chemotherapy drugs, including cisplatin, 5-fluoroutacil and paclitaxel are currently being explored in combination with sunitinib in clinical studies (22–24). Furthermore, in the present study, resistance of the endothelial cells to other agents used in targeted therapy was observed (data not shown). Ongoing clinical trials comprise numerous anti-angiogenic drugs (34,35). These trials require additional knowledge about the occurrence of cross-resistance, not only in tumor cells but also in endothelial cells.

Cross-resistance is complex as each ABC transporter can induce the efflux of a panel of chemical molecules based on physical affinity (18). Furthermore, a drug that consists of a single chemical can induce the upregulation of more than one ABC protein, as described in previous studies (12,20). During the establishment and optimization of a clinical therapeutic protocol with the provided therapeutic targets, it would be beneficial to evaluate and consider the potential risk of the development of cross-resistance to the treatment drugs. From this standpoint, additional effort would aid in the optimization of the combined use of chemotherapeutic agents and anti-angiogenic agents. Notably, as cross-resistance may occur in tumor and endothelial cells, it can also be speculated that the biological properties of drug resistance in these two types of cells may exhibit differences.

Since anti-angiogenic agents have been used clinically, patient resistance to anti-angiogenic drugs has been reported and analyzed frequently. Proposed mechanisms of resistance include alternative angiogenic escape factors, an increase in the stem cell population that is resistant to hypoxia, the selection of cells with acquired metastatic and invasive potential by hypoxia and tumor cell dormancy (11). Avoiding cross-resistance is expected to contribute to the further improvement of anticancer therapy, together with strategies that target multiple pathways involved in angiogenesis and resistance.

## Figures and Tables

**Table I:** Dox-induced cross-resistance to Dox and sunitinib in HMEC-1 endothelial cells.

	HMEC-1	HMECd1		HMECd2	
			
Agents	IC_50_, μM	IC_50_, μM	RI	IC_50_, μM	RI
Sunitinib	4.271±0.501	8.585±0.642	2.01^a^	19.252±0.855	4.51^a^
Doxorubicin	0.056±0.006	0.812±0.050	14.50^a^	1.209±0.085	21.60^a^

The cells were treated as described in the main text and tested with MTS assay. The RI was determined as the IC_50_ of Dox-treated HMECd1 or HMECd2 cells divided by the IC_50_ of Dox-treated HMEC-1 cells.

a P<0.05, vs. HMEC-1 cells.

Dox, doxorubicin; RI, resistance index; IC_50_, half maximal inhibitory concentration.

**Table II:** Effect of P-gp inhibitors and ABCG2 inhibitors on sunitinib resistance in HMEC-1 cells.

	HMEC-1	HMECd1			HMECd2		
			
Agents	IC_50_, μM	IC_50_, μM	RI	RF	IC_50_, μM	RI	RF
Sunitinib	4.271±0.501	8.585±0.642	2.01^a^	1.00	19.252±0.855	4.51^a^	1.00
+ 5 μM fumC	4.359±0.622	5.886±0.417	1.35^b^	1.45	6.163±0.062	1.41^b^	3.12
+ 0.5 μM die	4.204±0.468	6.259±0.541	1.48^b^	1.37	7.159±0.057	1.70^b^	2.69
+ 1 μM vrp	4.952±0.875	9.159±0.356	1.84	0.94	17.659±0.526	3.57	1.09
+ 2.5 μM cysA	4.098±0.562	8.871±0.459	2.16	0.97	20.348±0.328	4.97	0.95

The cells were treated as described in the main text and tested with MTS assay. The RI was determined by: IC_50_ of Dox-treated HMECd1 or HMECd2 cells/IC_50_ of Dox-treated HMEC-1 cells. The RF was calculated by: IC_50_ of Dox-treated HMECd1 or HMECd2 cells/IC_50_ of the same cell line treated by sunitinib + P-gp or ABCG2 inhibitors.

a P<0.05, vs. HMEC-1 cells;

b P<0.05, vs. HMECd1 and HMECd2 cells without P-gp or ABCG2 inhibitors.

P-gp, P-glycoprotein; ABCG2, breast cancer resistance protein; RI, resistance index; RF, resistance fold; IC_50_, half maximal inhibitory concentration; Dox, doxorubicin; fumC, fumitremorgin C; die, diethylstilbestrol; vrp, verapamil; cysA, cyclosporine A.

## Abbreviations

P-gp: P-glycoprotein
MRP1: multidrug resistance associated proteins
ABCG2: breast cancer resistance protein
qPCR: quantitative polymerase chain reaction
